# White Tea Modulates Metabolic Parameters and Adipokine Signaling in Experimental Obesity: Evidence for Functional Food Potential

**DOI:** 10.3390/ijms27094070

**Published:** 2026-05-01

**Authors:** Ayşegül Sümer, Öznur Demirtaş, Esra Pınarbaş Kanbur, Eda Yılmaz Kutlu, Mehtap Atak, Hülya Kılıç

**Affiliations:** Department of Medical Biochemistry, Faculty of Medicine, Recep Tayyip Erdogan University, 53100 Rize, Türkiye; oznur_demirtas19@erdogan.edu.tr (Ö.D.); esra.pinarbas@erdogan.edu.tr (E.P.K.); eda.yilmazkutlu@erdogan.edu.tr (E.Y.K.); mehtap.atak@erdogan.edu.tr (M.A.); hulya.kilic@erdogan.edu.tr (H.K.)

**Keywords:** white tea, functional food, obesity, bioactive compounds, adipokines, apelin, irisin, metabolic regulation

## Abstract

Functional foods enriched with bioactive compounds have attracted increasing attention for their potential to improve metabolic health and reduce the risk of chronic diseases. White tea, a minimally processed tea rich in polyphenols and antioxidant constituents, may exert beneficial effects on obesity-related metabolic disturbances through multiple molecular pathways. In this study, we investigated the effects of white tea in a high-fat diet-induced obesity model in rats, with particular emphasis on metabolic regulation and adipokine signaling. Body weight, lipid profile, glucose homeostasis, insulin resistance-related parameters, and circulating levels of apelin and irisin were evaluated. High-fat diet feeding impaired metabolic balance and altered obesity-associated biochemical parameters, whereas white tea administration ameliorated several of these changes. White tea was associated with improvements in body weight gain and selected metabolic parameters, together with modulation of adipokine-related markers. These findings suggest that white tea may function as a bioactive-rich functional food with beneficial effects on pathways involved in obesity and metabolic homeostasis. Our results support the potential contribution of white tea-derived compounds to nutrition-based strategies for the prevention and management of obesity.

## 1. Introduction

Obesity has become one of the most significant global public health challenges, with its prevalence increasing markedly over the past decades [1]. It is characterized by excessive adipose tissue accumulation resulting from a chronic imbalance between energy intake and expenditure [2,3]. Beyond its classical role as a depot for energy storage, adipose tissue is now recognized as a metabolically active endocrine organ that regulates systemic homeostasis through the secretion of numerous bioactive mediators, including adipokines, cytokines, and myokines [3,4]. Dysregulation of these signaling molecules contributes to metabolic disturbances such as insulin resistance, dyslipidemia, chronic low-grade inflammation, and impaired energy balance, thereby increasing the risk of type 2 diabetes, cardiovascular diseases, and other obesity-related complications [5,6].

Recent advances in nutrition and metabolism research have emphasized the importance of endocrine cross-talk between adipose tissue and other metabolically active organs in the maintenance of energy homeostasis. In this context, functional foods rich in bioactive compounds have attracted growing interest because of their ability to influence metabolic pathways at the molecular level and potentially reduce the risk of chronic disease. Among the regulatory pathways implicated in obesity, the apelin–insulin signaling axis and the irisin-related metabolic axis have emerged as particularly relevant [7,8].

Apelin is an endogenous peptide ligand of the G protein-coupled APJ receptor and is widely expressed in adipose tissue, skeletal muscle, and the cardiovascular system. It plays an important role in glucose metabolism, insulin sensitivity, cardiovascular regulation, and lipid homeostasis [9,10]. In adipose tissue, apelin functions as an adipokine capable of modulating glucose uptake and improving insulin-related signaling pathways. Several studies have reported elevated circulating apelin levels in individuals with obesity and metabolic syndrome, suggesting a compensatory response to metabolic stress [11,12]. However, growing evidence indicates that apelin regulation may vary according to the stage and severity of metabolic dysfunction, and early obesity-related alterations may suppress apelin expression, possibly reflecting impaired compensatory signaling within adipose tissue [13]. These findings highlight the complex role of apelin in metabolic homeostasis and support its relevance as a potential biomarker and therapeutic target in obesity.

In addition to adipokine signaling, irisin has attracted considerable attention as a mediator of metabolic adaptation. Irisin is a peptide hormone generated by the cleavage of fibronectin type III domain-containing protein 5 (FNDC5) and is involved in the endocrine communication between skeletal muscle and adipose tissue [14]. It has been associated with enhanced energy expenditure, metabolic regulation, and adaptive responses to metabolic stress. However, obesity and high-fat diet consumption are known to impair mitochondrial function and disrupt thermogenesis-related pathways, thereby reducing the efficiency of these protective responses [15,16,17]. In this regard, circulating irisin may serve as a valuable marker of obesity associated metabolic dysregulation.

Given the multifactorial nature of obesity, increasing interest has been directed toward food-derived bioactive compounds that may beneficially modulate metabolic pathways. Among these, tea derived from *Camellia sinensis* has been extensively investigated as a functional food due to its rich phytochemical composition and potential health-promoting effects. Tea contains a broad spectrum of bioactive compounds, particularly polyphenols such as catechins, epigallocatechin gallate (EGCG), flavonoids, and methylxanthines, which have been shown to exert antioxidant, anti-inflammatory, and anti-obesity activities [18,19,20]. Experimental evidence suggests that tea polyphenols can regulate lipid metabolism, promote fatty acid oxidation, activate AMP-activated protein kinase (AMPK), and improve insulin sensitivity [21,22]. These molecular effects support the growing recognition of tea and tea-derived products as promising dietary components in nutrition-based strategies for metabolic health.

White tea, produced from young leaves and subjected to minimal processing compared with other tea types, retains a high concentration of polyphenolic compounds and antioxidant constituents. Because of this distinctive phytochemical profile, white tea has gained increasing attention as a functional food with potential relevance for the prevention and management of metabolic disorders [23,24]. Previous experimental studies have suggested that white tea may reduce adipogenesis, improve lipid metabolism, and contribute to metabolic regulation in obesity models [25,26]. Nevertheless, the molecular mechanisms underlying these beneficial effects remain incompletely understood.

Importantly, although several studies have explored the impact of tea-derived polyphenols on obesity-related metabolic parameters, limited research has specifically examined the role of white tea in the regulation of adipokine-related signaling pathways in experimental obesity. Clarifying whether white tea can influence key metabolic mediators such as apelin and irisin may provide novel molecular insight into how functional foods contribute to metabolic homeostasis and obesity management.

Therefore, the present study aimed to investigate the metabolic effects of white tea in a high-fat diet-induced obesity model in rats by evaluating body weight changes, lipid and glucose metabolism, apelin-13 and apelin-36 levels, insulin resistance-related parameters, and circulating irisin levels. We hypothesized that white tea, as a bioactive-rich functional food, may be associated with favorable changes in metabolic parameters and circulating adipokine-related markers, thereby contributing to improved systemic metabolic homeostasis in diet-induced obesity.

## 2. Results

### 2.1. White Tea Supplementation Attenuated Body Weight Gain in Rats Fed a High-Fat Diet

**Figure 1:**
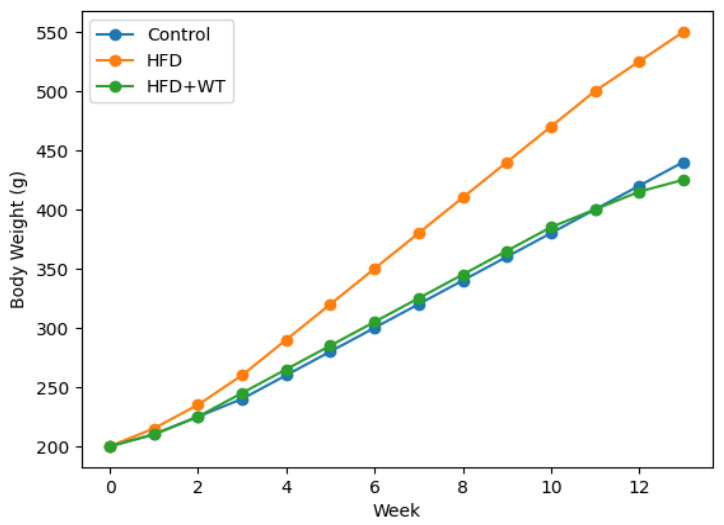
Weekly body weight changes in experimental groups during the 13-week study period.

At the end of the 13-week experimental period, the mean body weight gain rates were 53%, 82%, and 68% in the C (Control), HFD (High Fat Diet), and HFD+WT (High Fat Diet + White Tea) groups, respectively. The obesity model was considered successfully established, as the final body weight of the HFD group exceeded that of the control group by more than 20%. Moreover, final body weight differed significantly among the groups (Kruskal–Wallis test, χ^2^ = 18.340, *p* < 0.001), and post hoc analysis showed that the HFD group had significantly higher body weight than both the control and HFD+WT groups (Table 1, Figure 1).

### 2.2. Effects of White Tea on Glucose Metabolism

**Figure 2:**
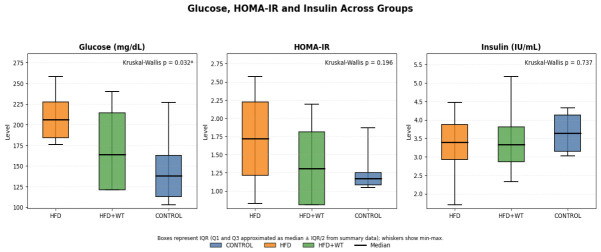
Serum insulin, fasting glucose and HOMA-IR values in experimental groups. Fasting glucose levels were significantly higher in the HFD group compared with the control group (* *p* < 0.05). No statistically significant differences were observed in insulin or HOMA-IR levels among the groups.

Fasting glucose, insulin, and Homeostatic Model Assessment of Insulin Resistance (HOMA-IR) levels were assessed to determine the metabolic effects of white tea in high-fat diet-fed rats. As presented in Figure 2, fasting glucose levels were significantly elevated in the HFD group compared with the control group (*p* < 0.05). In the HFD+WT group, fasting glucose levels were lower than those in the HFD group, indicating a partial attenuation of diet-induced impairment in glucose metabolism. In contrast, serum insulin and HOMA-IR values did not differ significantly among the groups. Collectively, these findings suggest that white tea may partially improve fasting glycemia in obese rats, although its effect on systemic insulin resistance appears limited under the present experimental conditions.

### 2.3. Effects on Lipid Metabolism

**Figure 3:**
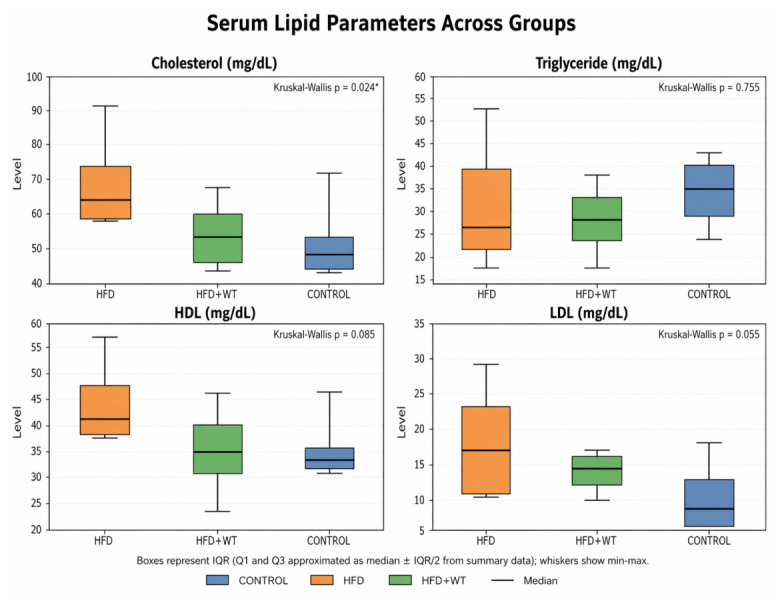
Effects of white tea on lipid metabolism in high-fat diet-fed rats. Total cholesterol, triglyceride, HDL-C, and LDL-C levels in experimental groups. Total cholesterol levels were significantly higher in the HFD group compared with the control group (* *p* < 0.05). No statistically significant differences were observed for triglyceride, HDL-C, or LDL-C levels among the groups.

Serum lipid parameters were assessed to evaluate the metabolic effects of white tea in rats fed a high-fat diet (Figure 3). Total cholesterol levels were significantly higher in the HFD group compared with the control group (*p* < 0.05). White tea supplementation was associated with lower cholesterol levels compared with the HFD group, indicating a potential improvement in lipid homeostasis. However, triglyceride (TG), high-density lipoprotein cholesterol (HDL-C), and low-density lipoprotein cholesterol (LDL-C) levels did not differ significantly among the experimental groups (*p* > 0.05).

### 2.4. Effects on Adipokine Levels

**Figure 4:**
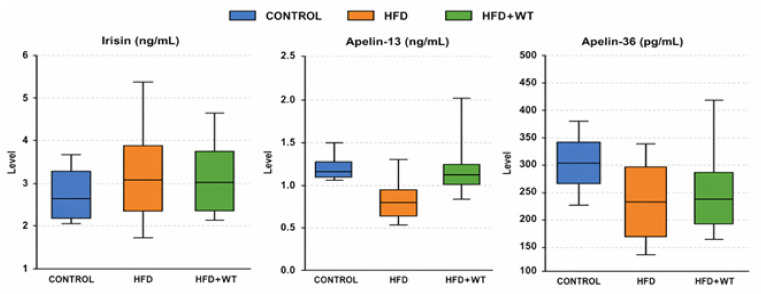
Effects of white tea on circulating adipokines in high-fat diet-fed rats.

Circulating adipokine levels were evaluated to investigate the metabolic effects of white tea in rats fed a high-fat diet (Figure 4). Serum irisin levels showed significant differences among the experimental groups, with higher levels observed in the HFD and white tea groups compared with the control group (*p* < 0.05). In contrast, apelin-13 levels were significantly reduced in the HFD group relative to the control group. White tea supplementation was associated with higher apelin-13 levels compared with the HFD group, suggesting a potential regulatory effect on adipokine homeostasis.

## 3. Discussion

In the present study, the metabolic effects of white tea supplementation were evaluated in a high-fat diet-induced obesity model by assessing body weight, glucose metabolism, lipid parameters, and adipokine levels. The main findings showed that high-fat diet feeding resulted in marked weight gain, together with elevated fasting glucose and total cholesterol levels compared with the control group. White tea supplementation attenuated body weight gain and was associated with partial improvement in selected metabolic parameters, particularly total cholesterol and apelin-13 levels. These findings support the view that white tea, as a bioactive-rich functional food, may exert beneficial effects on metabolic homeostasis under obesogenic conditions.

### 3.1. Phytochemical Characterization of White Tea and Its Functional Food Relevance

Among tea varieties, white tea has attracted increasing attention because it undergoes minimal processing and therefore retains substantial amounts of catechins and other phenolic compounds that may contribute to its metabolic effects. Recent reviews have highlighted that white tea, like green tea but with a partially distinct phytochemical profile, may exert anti-obesity actions through suppression of adipocyte differentiation, reduction in lipid accumulation, modulation of adipokine signaling, and improvement of obesity-related metabolic disturbances [27]. These effects are largely attributed to tea polyphenols, particularly EGCG, which has been reported to regulate lipid metabolism, improve insulin sensitivity, and attenuate ectopic fat accumulation in obesity-related conditions [28]. In addition, mechanistic evidence indicates that tea polyphenols can influence molecular pathways involved in adipogenesis, lipogenesis, lipolysis, and energy balance, thereby contributing to the regulation of adipose tissue function [29]. Although much of the literature has focused on green tea, long-term reviews and meta-analyses consistently support the broader concept that tea catechins can improve body composition, obesity-related hormonal responses, oxidative stress, and inflammatory status in metabolic disorders [30,31,32]. Collectively, these data provide a relevant framework for interpreting the findings of the present study and reinforce the potential of white tea as a functional food for metabolic health.

The HPLC characterization of the white tea sample provides an important biochemical context for interpreting the present findings. The analysis confirmed that the tea contained several bioactive compounds, particularly EGCG, total catechins, caffeine, and gallic acid. These constituents may provide a biochemical basis for the observed metabolic effects. EGCG and related catechins have been widely associated with improved metabolic regulation through multiple mechanisms, including attenuation of oxidative stress, suppression of low-grade inflammation, modulation of adipocyte differentiation, enhancement of fatty acid oxidation, and improvement of insulin-related signaling. These actions may partly explain the attenuation of body weight gain and the improvement in fasting glucose and total cholesterol levels observed in the white tea-treated group. In addition, catechin-rich tea bioactives may influence adipokine secretion by improving adipose tissue function and reducing metabolic stress, which may be relevant to the partial restoration of apelin-13 levels in the HFD+WT group. Caffeine, another major component detected in the white tea sample, may also contribute to energy expenditure and lipid mobilization.

Nevertheless, these interpretations should be considered mechanistic hypotheses rather than direct evidence. The present study did not administer isolated EGCG or individual catechins, nor did it perform correlation analyses between specific HPLC components and metabolic outcomes. Therefore, the observed effects are more likely attributable to the combined or synergistic actions of multiple white tea bioactives rather than to EGCG alone. Future studies using purified compounds, dose response designs, and tissue-level molecular analyses are needed to clarify the relative contribution of individual white tea constituents to metabolic regulation.

### 3.2. Effects of White Tea on Body Weight Regulation and Obesity-Related Metabolic Disturbance

One of the most prominent findings of the present study was the attenuation of body weight gain in rats receiving white tea supplementation despite ongoing exposure to a high-fat diet. The markedly higher final body weights observed in the HFD group confirmed the successful establishment of the diet-induced obesity model. In contrast, white tea treatment significantly reduced weight gain relative to the HFD group, suggesting that it may counteract some of the obesogenic effects of high-fat feeding.

Previous studies supported the anti-obesity potential of white tea. Clinical investigations have reported that regular white tea consumption may improve anthropometric measurements and metabolic markers in obese individuals [33]. Similarly, experimental studies in rodent models have shown that white tea administration attenuates body weight gain and improves metabolic performance under obesogenic conditions [34]. In a high-fat diet-induced obesity model, white tea supplementation was reported to significantly reduce body weight and modulate adipokine levels [35]. At the cellular level, white tea extracts have been shown to inhibit adipocyte differentiation and stimulate lipolysis in adipose tissue models [36]. Taken together, these findings support the hypothesis that white tea may influence body weight regulation through effects on adipogenesis, lipid metabolism, and endocrine signaling pathways relevant to obesity.

### 3.3. Effects of White Tea on Glucose and Lipid Metabolism

White tea supplementation was also associated with changes in glucose metabolism. In the present study, fasting glucose levels were significantly higher in the HFD group than in the control group, indicating impaired glucose homeostasis after high-fat diet feeding. In contrast, fasting glucose levels were lower in white tea-treated animals than in the untreated HFD group. However, insulin and HOMA-IR values did not differ significantly among the groups. These findings suggest that white tea supplementation may improve fasting glucose control without inducing marked changes in circulating insulin concentrations.

Previous experimental evidence is generally consistent with this observation. Daily white tea consumption has been reported to affect glucose-related metabolic pathways and improve metabolic profiles in prediabetic rat models [37]. In streptozotocin-induced diabetic rats, white tea extracts significantly reduced fasting blood glucose levels compared with untreated diabetic controls [38]. Likewise, long-term white tea consumption has been shown to attenuate high-fat diet-induced alterations in glucose metabolism and reduce elevated blood glucose levels in experimental animals [39]. Clinical studies have also suggested that regular white tea intake may contribute to improved metabolic parameters in obese individuals [33]. Together, these data indicate that white tea-derived bioactive compounds beneficially influence glucose homeostasis, although the extent of this effect may vary depending on the experimental model and the metabolic variables examined.

Lipid metabolism represents another important domain affected by high-fat diet feeding. In the present study, total cholesterol levels were significantly elevated in the HFD group and were reduced following white tea administration. In contrast, triglyceride, HDL-C, and LDL-C levels did not show statistically significant differences among the groups. These findings indicate that white tea supplementation was associated with selective improvement in lipid metabolism, particularly at the level of total cholesterol, whereas other lipid fractions remained relatively unchanged in this model.

The pattern of significant and non-significant metabolic findings should be interpreted cautiously. Although fasting glucose and total cholesterol levels were significantly altered by high-fat diet feeding, insulin, HOMA-IR, TG, HDL-C, and LDL-C did not differ significantly among the groups. These non-significant findings may be explained by several factors. First, the high-fat diet model used in this study may represent an early or moderate stage of metabolic dysfunction in which fasting hyperglycemia and cholesterol changes occur before the development of marked hyperinsulinemia or insulin resistance. Second, circulating insulin and HOMA-IR values are influenced by compensatory pancreatic β-cell responses and may not fully reflect tissue-specific insulin sensitivity. Third, serum lipid fractions do not necessarily change in parallel; total cholesterol may be more responsive to dietary fat intake and white tea supplementation, whereas TG, HDL-C, and LDL-C may show greater biological variability and may require longer intervention periods, larger sample sizes, or tissue-level lipid metabolism analyses to detect significant differences. Therefore, the absence of significant changes in these parameters should be interpreted cautiously and does not exclude the possibility of tissue-specific metabolic effects of white tea.

The cholesterol-lowering effect observed in the present study is supported by previous reports showing that white tea supplementation can improve certain lipid-related parameters in experimental obesity [35]. Our findings are also consistent with previous experimental studies reporting that white tea extracts can affect adipose tissue-related processes at the cellular level. Söhle et al. demonstrated that white tea extract suppresses adipogenesis and enhances lipolysis in human subcutaneous (pre)adipocytes, accompanied by downregulation of PPARγ, ADD1/SREBP-1c, C/EBPα, and C/EBPδ [40]. This white tea-specific evidence is consistent with broader contemporary reviews indicating that tea bioactives may exert anti-obesity effects through coordinated actions on oxidative stress, inflammation, glucose metabolism, and adipocyte regulation [41]. In parallel, He et al. summarized that polyphenol-mediated anti-obesity activity is frequently associated with inhibition of adipogenic transcriptional programs, activation of AMPK-related metabolic signaling, improved insulin sensitivity, and enhanced energy expenditure-related responses [29]. Together, these reports provide biological plausibility for the metabolic changes observed after white tea administration in the present study, while direct mechanistic confirmation requires further tissue-level and pathway-focused analyses.

### 3.4. Changes in Circulating Adipokine-Related Markers: Apelin and Irisin

A major objective of this study was to evaluate the effects of white tea on circulating adipokine-related markers. Apelin is an adipose tissue-derived peptide with reported roles in glucose metabolism, insulin-related responses, and lipid homeostasis [24]. In the present study, apelin-13 levels were significantly reduced in rats fed a high-fat diet compared with controls, suggesting that high-fat feeding was associated with altered circulating apelin-13 concentrations. White tea supplementation was associated with higher apelin-13 levels than those observed in the HFD group. These findings suggest that white tea may be related to changes in circulating adipokine profiles in experimental obesity; however, they do not establish a direct effect on apelin/APJ signaling.

Apelin circulates in several biologically active isoforms, including apelin-13 and apelin-36, which may differ in biological potency, stability, receptor interaction, and metabolic responsiveness. Apelin-13 is generally considered a highly active isoform and has been associated with glucose uptake, insulin sensitivity, cardiovascular regulation, and adipose tissue function. In the present study, apelin-13 levels were significantly reduced in the HFD group and were higher in the white tea group, whereas apelin-36 did not differ significantly among the groups. This pattern may indicate that circulating apelin-13 is more responsive than apelin-36 to diet-induced metabolic changes and white tea supplementation in this experimental setting. However, because tissue apelin expression and APJ receptor activation was not assessed, this interpretation remains preliminary and should not be considered evidence of direct apelinergic pathway modulation.

Another notable finding was the alteration in circulating irisin levels. Irisin is a peptide hormone associated with systemic metabolic regulation, energy balance, and adaptive responses to metabolic stress [42]. In this study, circulating irisin levels were significantly higher in both the HFD and white tea groups than in the control group. These data indicate that high-fat diet feeding was associated with increased irisin levels, while white tea supplementation did not reverse this increase. Rather than reflecting a direct normalization effect, this pattern may suggest that elevated irisin represents an adaptive or compensatory endocrine response to metabolic stress induced by high-fat feeding.

Recent studies increasingly emphasize the importance of inter-organ endocrine communication in metabolic regulation. Although apelin and irisin originate predominantly from different tissues, both are involved in the maintenance of systemic metabolic homeostasis [43,44]. Apelin is primarily secreted by adipose tissue and contributes to the regulation of glucose metabolism and energy balance, whereas irisin is mainly released from skeletal muscle and has been linked to metabolic adaptation and whole-body energy regulation [45,46]. Emerging evidence suggests that adipose tissue and skeletal muscle communicate through endocrine signaling networks that coordinate substrate utilization and energy homeostasis [32,47]. In this context, simultaneous evaluation of apelin and irisin may provide a more integrated view of obesity associated endocrine dysregulation. The present findings therefore contribute to a better understanding of how white tea, as a functional food rich in bioactive compounds, may interact with multiple regulatory systems involved in metabolic health.

### 3.5. Limitations and Future Perspectives

Several limitations of the present study should be acknowledged. First, although 24 rats were initially allocated equally across the three experimental groups, one animal in the control group was lost before the final analysis, resulting in a final sample size of seven animals in this group. Although this minor imbalance is unlikely to have substantially altered the overall interpretation of the findings, the relatively small sample size may have reduced the statistical power to detect subtle differences, particularly in parameters with high biological variability, such as insulin, HOMA-IR, triglycerides, HDL-C, and LDL-C.

Second, the study was conducted in a rat model of high-fat diet-induced obesity; therefore, the findings cannot be directly extrapolated to humans. Species-specific differences in metabolism, adipose tissue biology, hormonal regulation, gastrointestinal absorption, and polyphenol bioavailability may influence the biological effects of white tea. In addition, only male rats were used, which may limit the generalizability of the findings, since sex-related differences in adipose tissue distribution, endocrine responses, and metabolic regulation may affect the response to dietary bioactives.

Third, white tea was administered by oral gavage. This route allowed controlled and reproducible dosing but does not fully mimic habitual tea consumption in humans. Oral gavage may also influence stress responses, absorption dynamics, feeding behavior, and the interaction of tea bioactives with meals. Therefore, future studies using voluntary consumption or dietary incorporation models may provide more translationally relevant information.

Fourth, only a single dose of white tea was administered. Therefore, the dose–response relationship and the optimal effective dose could not be determined. Although the selected dose was based on previous experimental evidence, studies using multiple dose levels are needed to clarify whether the metabolic effects of white tea are dose dependent.

Fifth, the present study focused mainly on circulating metabolic and adipokine-related markers. Tissue-specific molecular analyses, including AMPK, PPARγ, FNDC5, apelin/APJ receptor expression, thermogenic markers, oxidative stress markers, inflammatory mediators, or pathway-specific protein activation, were not performed. Therefore, the observed changes should be interpreted as systemic metabolic and endocrine responses rather than direct evidence of specific intracellular signaling mechanisms.

Sixth, although HPLC characterization identified major bioactive constituents of white tea, including EGCG, total catechins, caffeine, and gallic acid, the independent effects of these compounds were not evaluated. Therefore, direct causal links between individual phytochemicals and metabolic outcomes cannot be established. The observed effects are more likely attributable to the combined or synergistic actions of multiple white tea bioactives rather than to EGCG alone.

Seventh, apelin was evaluated only at the circulating level, and tissue apelin expression or APJ receptor signaling was not assessed. Therefore, the isoform-specific differences observed between apelin-13 and apelin-36 should be interpreted cautiously. Further studies are required to clarify whether these differences reflect isoform stability, receptor interaction, tissue-specific regulation, or metabolic responsiveness.

Finally, the absence of significant changes in insulin, HOMA-IR, TG, HDL-C, and LDL-C should be interpreted with caution. These findings may reflect the relatively early or moderate stage of metabolic impairment in the present model, compensatory insulin regulation, biological variability in circulating lipid fractions, or limited statistical power.

From a future perspective, larger and longer-term studies are needed to more comprehensively clarify the metabolic effects of white tea in obesity-related dysfunction. Future experimental designs should include multiple white tea doses, both sexes, voluntary consumption or dietary incorporation models, and broader tissue-level analyses in adipose tissue, liver, skeletal muscle, and hypothalamus. In addition, studies evaluating AMPK, PPARγ, FNDC5/irisin, apelin/APJ signaling, thermogenic markers, oxidative stress, and inflammatory pathways at both gene and protein levels would help define the molecular mechanisms underlying the observed effects. Further investigations using isolated tea bioactives, such as EGCG, caffeine, and gallic acid, as well as combined bioactive formulations, may also clarify whether the beneficial effects of white tea result from individual compounds or synergistic interactions. Ultimately, well-designed translational and clinical studies are required to determine whether white tea can be considered a practical functional food strategy for the nutritional management of obesity and metabolic health.

### 3.6. Conclusions

In conclusion, white tea supplementation attenuated body weight gain and was associated with improvement in selected metabolic parameters in a high-fat diet-induced obesity model. White tea treatment was linked to lower fasting glucose and total cholesterol levels compared with the untreated high-fat diet group. In addition, changes in circulating adipokine-related markers were observed, including increased apelin-13 concentrations, while elevated irisin levels persisted in animals exposed to a high-fat diet. Overall, these findings suggest that white tea may be associated with favorable systemic metabolic and endocrine responses in experimental obesity. However, because tissue-level molecular pathways and receptor-mediated signaling were not directly assessed, the present results should be interpreted as biochemical and endocrine marker changes rather than direct mechanistic evidence. The study adds to the growing body of evidence supporting white tea as a promising functional food candidate for further investigation in the context of obesity and metabolic health.

## 4. Materials and Methods

### 4.1. Chemicals

White tea used in the study was obtained from the General Directorate of Tea Enterprises (ÇAYKUR, Rize, Türkiye). For biochemical analyses, rat apelin-13 ELISA kit (Cat. No. E1427Ra, Lot: 202103014), rat apelin-36 ELISA kit (Cat. No. E1331Ra, Lot: 202103014), rat insulin ELISA kit (Cat. No. E0707Ra, Lot: 202103014), and rat irisin ELISA kit (Cat. No. E6281Ra) were obtained from BT Lab/Bioassay Technology Laboratory, Shanghai Korain Biotech Co., Ltd. (Shanghai, China), and used according to the manufacturer’s instructions. A high-fat diet containing 22% of total energy from fat was obtained from Arden Research & Experiment (Ankara, Türkiye) (Table 1). For anesthesia during experimental procedures, ketamine (Ketalar^®^, 100 mg/10 mL, Pfizer, New York, NY, USA) and xylazine hydrochloride (Rompun^®^, 2%, 25 mL, Bayer, Leverkusen, Germany) were administered.

### 4.2. Animals and Housing

This study was approved by the Local Ethics Committee for Animal Experiments at Recep Tayyip Erdoğan University (RTEU) (protocol no: 2020/39; approved on 26 June 2020) and was conducted in accordance with the ARRIVE 2.0 guidelines. A total of 24 male Sprague Dawley rats (6 weeks old) were obtained from the RTEU Experimental Animal Research and Application Center. Animals were housed under controlled environmental conditions (23 ± 2 °C, 55 ± 5% relative humidity, and a 12 h light/dark cycle) in transparent polyethylene cages with sawdust bedding.

Following one week of acclimatization with a standard pellet diet (Bayramoğlu Yem ve Un San. Tic. A.Ş., Full Pellet Rat Feed) and water provided ad libitum, rats were randomly allocated to three experimental groups (*n* = 8 per group) using a simple randomization method. The sample size was determined based on previous experimental studies investigating diet-induced obesity models in rodents.

To induce obesity, animals in the high-fat diet groups were fed a high-fat diet (HFD; 22% kcal from fat; Arden Research & Experiment) ad libitum at approximately 15–20 g per rat per day. Water was provided using specialized drinking bottles.

### 4.3. Experimental Design

To evaluate the protective effects of white tea, the rats were divided into three groups:

- Control group (C)—standard pellet diet;
- High-fat diet group (HFD)—high-fat diet;
- High-fat diet + white tea group (HFD+WT)—high-fat diet plus white tea treatment.

White tea was administered to the HFD+WT group at a dose of 5 mg/kg body weight/day by oral gavage for 12 weeks. The control and HFD groups received tap water by oral gavage twice per week to ensure comparable handling conditions.

Body weights were recorded weekly throughout the experimental period, and the administered dose of white tea was adjusted according to body weight changes. The experimental period lasted 12 weeks, during which the obesity model was successfully established in HFD-fed animals. The obesity model was considered established when the body weight of rats in the HFD group exceeded that of the control group by more than 20% [48].

Investigators responsible for biochemical measurements and statistical analyses were blinded to group allocation to minimize potential bias.

### 4.4. Sample Collection

Prior to sample collection, animals were fasted overnight to standardize metabolic conditions. On the day of the procedure, deep surgical anesthesia was induced via intraperitoneal administration of a ketamine–xylazine mixture (3:1). Adequate anesthetic depth was confirmed by the absence of corneal and pedal withdrawal reflexes in accordance with ethical and veterinary guidelines.

Blood samples were collected via intracardiac puncture from the left ventricle using sterile syringes. Animals were subsequently euthanized by exsanguination while under deep anesthesia.

At the end of the experiment, one rat from the control group was lost, and statistical analyses were performed using the remaining data.

### 4.5. Tea Preparation

White tea (WT) leaves were obtained from the General Directorate of Tea Enterprises (Rize, Türkiye). The leaves were harvested from Camellia sinensis plants in May 2020. The brewing procedure was performed according to a previously reported method [34]. Briefly, the required amount of tea leaves was weighed and infused in water that had been boiled at 100 °C and allowed to cool slightly to 97–98 °C. The infusion was covered and brewed for 10 min. This temperature and brewing duration were selected due to the high antioxidant potential of white tea [49].

During the study, WT was stored in a dark and dry environment under appropriate conditions. The tea was freshly prepared each day, kept at room temperature, and administered to the rats to avoid potential antioxidant loss. Body weights were measured weekly, and the administered doses were recalculated accordingly. Obesity was defined as a ≥20% increase in body weight in the experimental groups compared with the control group [49].

### 4.6. Determination of White Tea Bioactive Components by HPLC

The phytochemical composition of tea prepared using a similar method has been previously characterized in a previous study [35]. In the present study, the bioactive composition of the white tea sample was further characterized by high-performance liquid chromatography (HPLC), and the results are presented in Table 2.

### 4.7. Biochemical Experiments

Animals were fasted overnight prior to sample collection. On the day of the procedure, deep surgical anesthesia was induced by intraperitoneal administration of a ketamine–xylazine hydrochloride mixture at a 3:1 ratio. Adequate anesthetic depth was confirmed by the absence of pedal and corneal reflexes. Blood samples were then collected via intracardiac puncture from the left ventricle using sterile syringes. While still under deep anesthesia, the animals were humanely euthanized.

Blood samples were centrifuged at 3000 rpm for 15 min at 4 °C to obtain serum. The separated serum samples were stored at −80 °C until biochemical analyses to prevent protein degradation.

Serum levels of apelin-13, apelin-36, insulin, and irisin were measured using commercially available enzyme-linked immunosorbent assay (ELISA) kits according to the manufacturers’ instructions. Briefly, serum samples and standards were added to antibody-coated 96-well microplates. After incubation and washing steps, a biotinylated detection antibody, followed by a streptavidin–HRP conjugate, was applied. The enzymatic reaction was initiated by adding the substrate solution, and the reaction was terminated after color development. Optical density (OD) was measured at 450 nm using a microplate reader, and concentrations were calculated based on the corresponding standard curves.

Serum triglyceride (TG), total cholesterol (TC), high-density lipoprotein cholesterol (HDL-C), low-density lipoprotein cholesterol (LDL-C) levels, and fasting blood glucose (FBG) were measured using an automated biochemical analyzer (Beckman Coulter AU5800).

Insulin resistance was assessed using the Homeostatic Model Assessment for Insulin Resistance (HOMA-IR) according to the following formula:

HOMA-IR = Fasting Insulin (μU/mL) × Fasting Glucose (mg/dL)/405

### 4.8. Statistical Analysis

Statistical analyses were carried out using IBM SPSS Statistics version 25.0 (IBM Corp., Armonk, NY, USA). The normality of numerical variables was evaluated based on skewness and kurtosis values and values within ±3 was accepted as indicating approximate normality. However, since normality was not satisfied for all variables and the group sizes were limited, non-parametric tests were preferred.

Differences among the Control, HFD, and HFD+WT groups were analyzed using the Kruskal–Wallis H test. When the overall test result was significant, pairwise comparisons were performed using Bonferroni-adjusted post hoc analysis. Because three pairwise comparisons were conducted, the corrected level of significance was accepted as *p* < 0.0167. Data are presented as median and interquartile range (IQR, 25th–75th percentiles).

## Figures and Tables

**Table 1:** Final body weights of the groups.

Parameter	Group	*n*	Median	IQR	X^2^	*p*
Body weight (g)	HFD	8	549.50	23.50	18.340	0.000 *
	HFD+WT	8	423.50	44.75		
	C	7	460.00	50.00		

IQR, interquartile range; X^2^, Kruskal–Wallis test statistic; HFD, high-fat diet group; HFD+WT, high-fat diet plus white tea group. Data are presented as median and interquartile range (IQR). Differences among groups were analyzed using the Kruskal–Wallis test. X^2^ indicates the Kruskal–Wallis test statistic. * *p* < 0.05 indicates statistical significance.

**Table 2:** HPLC-based characterization of white tea bioactive components.

Component	% Dry Matter	% Dry Matter *
Gallic acid	0.11	0.18
Caffeine	5.01	4.83
EGC	0.38	0.00
C	0.00	0.06
EC	0.87	0.33
EGCG	9.20	4.75
ECG	2.28	1.07
Total Catechin (EGC + EC + EGCG + ECG)	12.74	6.21

EGC, epigallocatechin; C, catechin; EC, epicatechin; EGCG, epigallocatechin gallate; ECG, epicatechin gallate. The analysis was performed at the ÇAYKUR Agricultural Research Laboratory using the ISO 14502-2:2005 standard method. * Values in this column represent the phytochemical composition of the white tea infusion prepared by adding 1.5 g of white tea to boiled pure water and brewing for 10 min. The concentrations of gallic acid, caffeine, and major catechin derivatives were calculated on a dry matter basis (% dry matter).

## Data Availability

The data presented in this study is available on request from the corresponding author.

## Abbreviations

The following abbreviations are used in this manuscript:

Abbreviation	
ADD1/SREBP-1c	Adipocyte Determination and Differentiation Factor 1/Sterol Regulatory Element-Binding Protein-1c
AMPK	AMP-Activated Protein Kinase
APJ	Apelin Receptor
ARRIVE	Animal Research: Reporting of In Vivo Experiments
C/EBPα	CCAAT/Enhancer-Binding Protein Alpha
C/EBPδ	CCAAT/Enhancer-Binding Protein Delta
EGCG	Epigallocatechin Gallate
ELISA	Enzyme-Linked Immunosorbent Assay
FNDC5	Fibronectin Type III Domain-Containing Protein 5
FBG	Fasting Blood Glucose
HDL-C	High-Density Lipoprotein Cholesterol
HOMA-IR	Homeostatic Model Assessment for Insulin Resistance
HRP	Horseradish Peroxidase
IBM	International Business Machines
IQR	Interquartile Range
LDL-C	Low-Density Lipoprotein Cholesterol
OD	Optical Density
PPARγ	Peroxisome Proliferator-Activated Receptor Gamma
SD	Standard Deviation
SPSS	Statistical Package for the Social Sciences
TC	Total Cholesterol
TG	Triglyceride
WT	White Tea
